# The biological mechanism and emerging therapeutic interventions of liver aging

**DOI:** 10.7150/ijbs.87679

**Published:** 2024-01-01

**Authors:** Wenchao Wang, Kangdi Xu, Mingge Shang, Xiang Li, Xinyu Tong, Zhengtao Liu, Lin Zhou, Shusen Zheng

**Affiliations:** 1 Division of Hepatobiliary and Pancreatic Surgery, Department of Surgery, First Affiliated Hospital, School of Medicine, Zhejiang University, Hangzhou 310003, China.; 2 NHC Key Laboratory of Combined Multi-organ Transplantation, Hangzhou 310003, China.; 3 Key Laboratory of the diagnosis and treatment of organ Transplantation, Research Unit of Collaborative Diagnosis and Treatment for Hepatobiliary and Pancreatic Cancer, Chinese Academy of Medical Sciences (2019RU019), Hangzhou 310003, China.; 4 Key Laboratory of Organ Transplantation, Research Center for Diagnosis and Treatment of Hepatobiliary Diseases, Zhejiang Province, Hangzhou 310003, China.; 5 Shulan International Medical College, Zhejiang Shuren University, Hangzhou 310015, Zhejiang, China.; 6 Shulan (Hangzhou) Hospital Affiliated to Zhejiang Shuren University Shulan International Medical College, Hangzhou 310000, China.

**Keywords:** Liver aging, Multi-omics analysis, Model of liver aging, Therapeutic approach.

## Abstract

Research on liver aging has become prominent and has attracted considerable interest in uncovering the mechanism and therapeutic targets of aging to expand lifespan. In addition, multi-omics studies are widely used to perform further mechanistic investigations on liver aging. In this review, we illustrate the changes that occur with aging in the liver, present the current models of liver aging, and emphasize existing multi-omics studies on liver aging. We integrated the multi-omics data of enrolled studies and reanalyzed them to identify key pathways and targets of liver aging. The results indicated that C-X-C motif chemokine ligand 9 (Cxcl9) was a regulator of liver aging. In addition, we provide a flowchart for liver aging research using multi-omics analysis and molecular experiments to help researchers conduct further research. Finally, we present emerging therapeutic treatments that prolong lifespan. In summary, using cells and animal models of liver aging, we can apply a multi-omics approach to find key metabolic pathways and target genes to mitigate the adverse effects of liver aging.

## Background

Senescent cells experience growth arrest, increased autophagy, metabolic reprogramming, the implementation of a complex proinflammatory secretome, and chromatin remodeling 1. Hayflick et al. first described cellular senescence 60 years ago when they discovered that human diploid fibroblasts have a limited replicative potential in culture, after which they enter a state of irreversible replicative arrest 2. This phenomenon was labeled replicative senescence, and it is related to the constant shortening of telomere DNA during cell division. Finally, cell division is precluded. This is known as the “telomere theory of senescence” 3. In addition, cyclin-dependent kinase inhibitor 2A (P16) and tumor protein p53 (P53) are upregulated, and the cell cycle-promoting genes E2F transcription factor 8 (E2F8) and transcription factor Dp-1 (TFDP1) are downregulated, in senescent cells 4, 5.

The aged liver exhibits many changes in morphology and structure 6. Macroscopically, the aged liver is reduced in size and blood flow 7, 8. Among hepatocytes, the smooth endoplasmic reticulum is lost, the number of mitochondria is reduced together with an increase in volume, the volume of the dense body compartment is enlarged, and polyploidy is enhanced 9. Biological processes are changed in aged livers, leading to the development of age-associated liver diseases. The incidence of high triglyceride levels, nonalcoholic fatty liver diseases, and hepatocellular carcinoma increases with liver aging 10-12. However, the most dramatic effect of liver aging is its compromised ability to regenerate after loss of mass from surgical or chemical injury.

Various kinds of stress responses, such as the unfolded protein response and responses to reactive oxygen species (ROS), DNA-damaging agents, and radiation, are all contributors to cell senescence 3, 13. As such, there exist some methods that can induce aging, including D-galactose (D-gal) injection, hydrogen peroxide (H2O2) induction, and X-ray induction, which can commonly be used to induce cell or rat aging 14, 15. Azman et al. summarized the effects of D-gal on liver aging and its underlying mechanisms, including ROS generation, oxidative stress induction, and an increased inflammatory response, which in turn lead to mitochondrial dysfunction, apoptosis, and hepatic structural damage 16.

Currently, liver transplantation (LT) is considered one of the most important curative treatments for end-stage liver disease. In light of the growing number of patients on the waiting list for LT, the use of marginal grafts has also been maximized to expand the donor pool 17. Hence, an increasing number of grafts from older donors are being used in LT. However, the antioxidant and regenerative abilities decline with liver aging, livers from old donors suffer more severe ischemia-reperfusion injury (IRI), and the recovery ability of the liver after transplantation is poor, which affects the success of LT. Hence, advanced donor age is a contributor to poor quality of the liver, and there is a high incidence of graft loss and complications, including ischemic-type biliary complications and hepatic artery thrombosis, following LT from older donors 18. Hence, it is necessary for clinicians to discover new therapeutic interventions to combat liver aging that remove or mitigate the known adverse effects to improve graft survival after LT.

**Figure 1:**
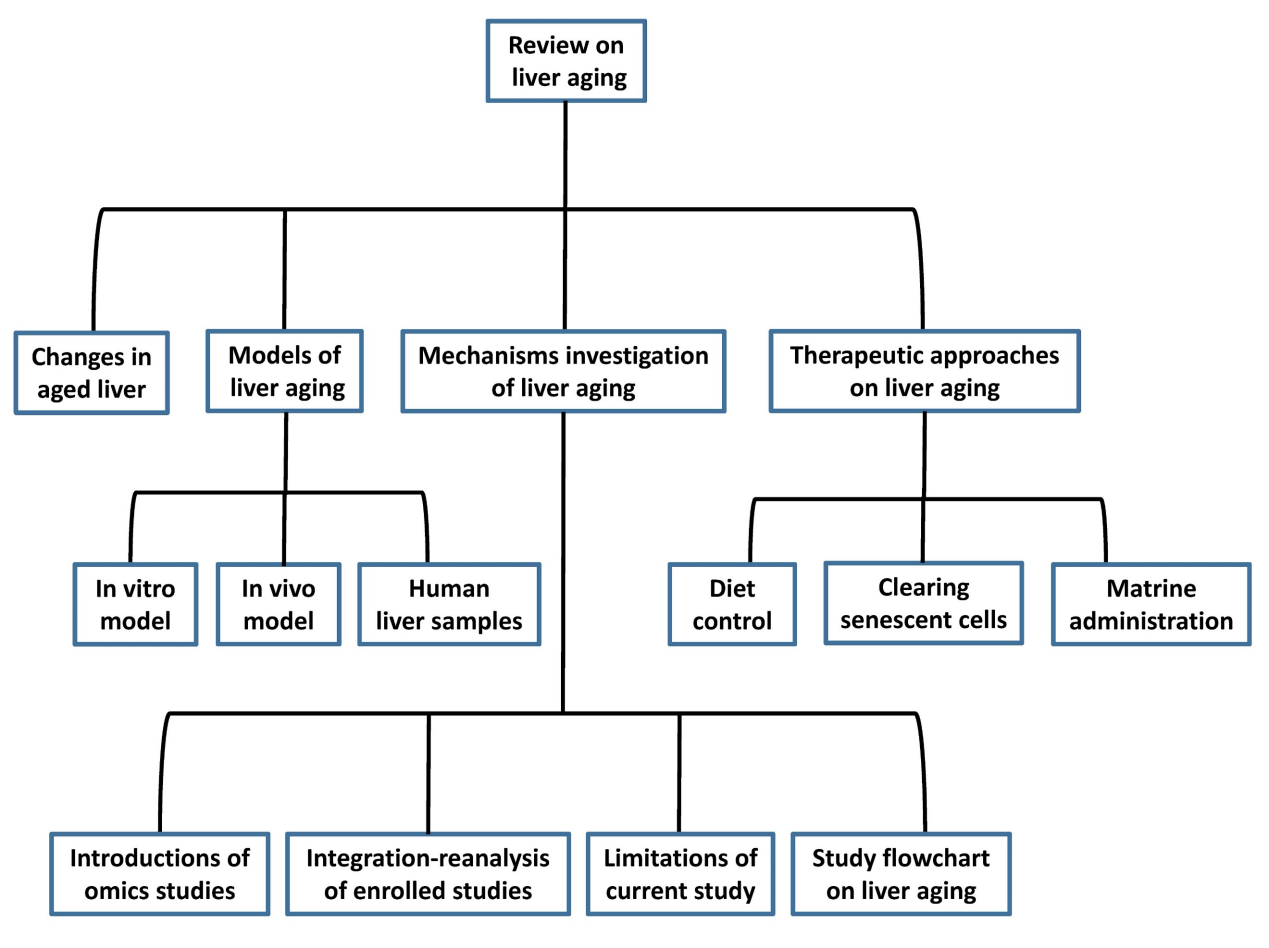
Flow chart of the study design.

In this manuscript, we discuss liver aging in detail and introduce the present models for mimicking the aged liver. We also summarize the mechanisms and pathways of liver aging and provide insights into the study of liver aging using multi-omics analysis and molecular experiments based on models of liver aging. Finally, we review the therapeutic interventions reported by published articles. The flowchart of the study is in Figure 1. We hope that this review can help researchers design antiaging therapies to improve the quality of the aged liver.

## Main text

### What is liver aging?

**Figure 2:**
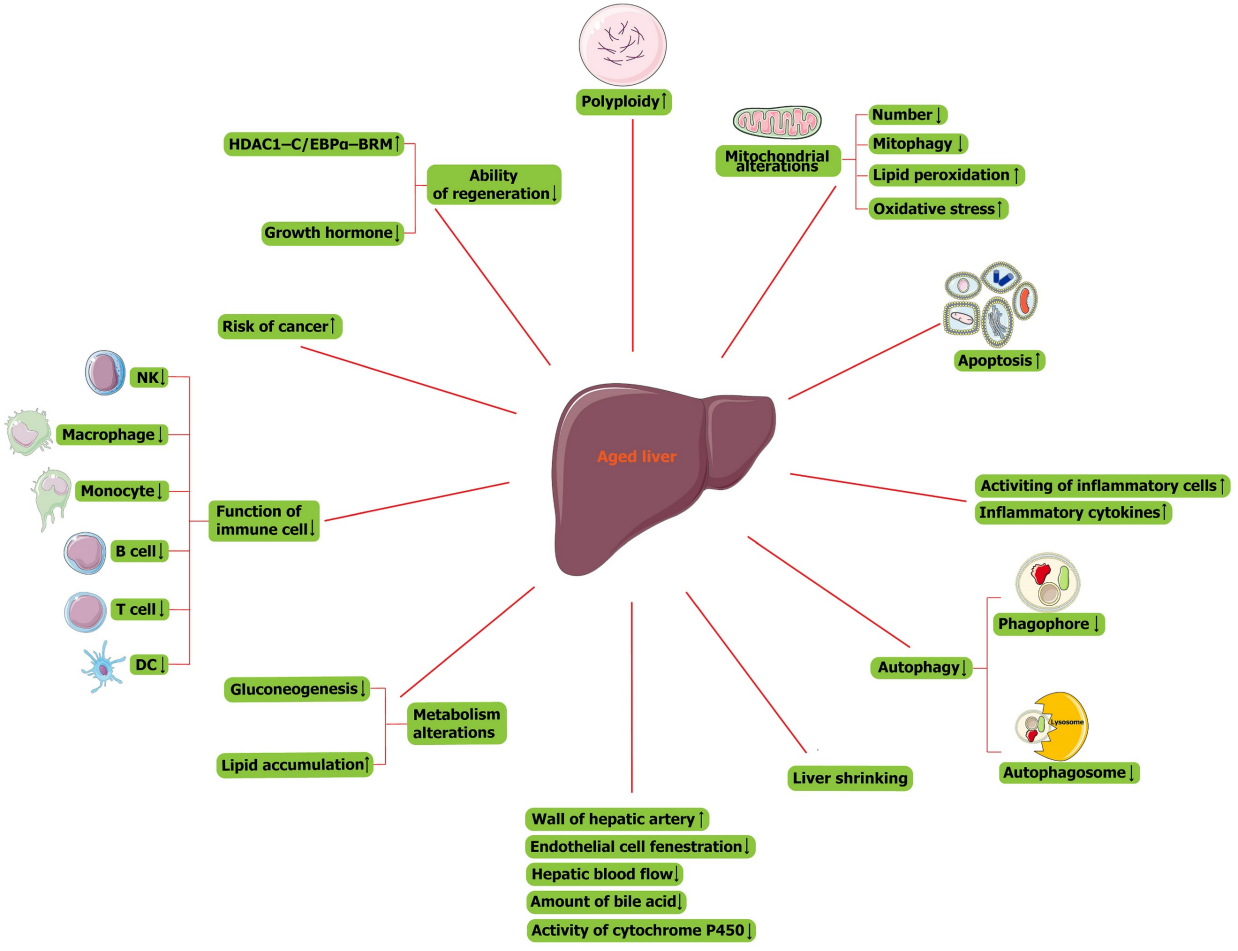
Main changes in the liver with physiological aging. NK cell, natural killer cell; DC, dendritic cell; HDAC1, histone deacetylase 1; C/EBPα, CCAAT/enhancer-binding protein (C/EBP) α; BRM, SWI/SNF related, matrix associated, actin dependent regulator of chromatin, subfamily a, member 2.

The signature of cellular senescence includes the presence of permanent cell cycle arrest, acquisition of major morphological changes, expression of senescence-associated beta-galactosidase (SA-β-gal), accumulation of senescence-associated heterochromatic foci (SAHF), senescence-associated DNA damage foci (SADF), acquisition of a senescence-associated secretory phenotype (SASP), increased ROS production, and autophagy 19-21. Main changes in the aged liver were presented in Figure 2. At the cellular level, age-related liver changes present as an increase in the size of hepatocytes, which relates to the observed increase in polyploidy (Figure 2). Polyploid cells account for 6%-15% of hepatocytes in 20-year-old adults and 25%-42% of hepatocytes in 80-year-old adults 22, 23. Therefore, polyploidy is considered a sign of cellular senescence and a stress response to limit the proliferation of damaged cells 24, 25. In addition, the number of mitochondria and mitochondrial mitophagy are decreased (Figure 2). Mitochondrial dysfunction of senescent cells leads to DNA damage and lipid peroxidation, further causing a vicious cycle of increased levels of oxidative stress and increased cell sensitivity to this stress (Figure 2) 26. Although apoptosis increases in the aged liver (Figure 2) and studies have reported that the apoptosis of senescent cells is helpful for liver homeostasis 27, senescent cells are generally considered resistant to apoptosis 28. In addition, the aged liver is more susceptible to fibrosis. A significant number of inflammatory cells are activated, and inflammatory cytokine levels are also increased (Figure 2) 29. In phagocytes, the number of phagophores and autophagosomes is decreased, so autophagy is decreased (Figure 2). All these factors contribute to the tendency toward liver fibrosis 30.

There are many morphological and functional changes in the aged liver. As the liver ages, its size shrinks by approximately 20%-40% (Figure 2), and this change is more pronounced in women than in men 26. In addition, the wall of the hepatic artery becomes thicker, endothelial cell fenestration is reduced, and the hepatic blood flow and amount of bile acid secreted by hepatocytes are decreased (Figure 2). Furthermore, the activity of cytochrome P450 is also reduced in senescent hepatocytes (Figure 2) 31. Broadly, the metabolism of the liver is altered with aging: the hepatic capacity for gluconeogenesis is decreased, and more lipids accumulate (Figure 2), so steatosis occurs in the aged liver 32-34. In addition, the immune system becomes abnormal in the aged liver (Figure 2). The function of immune cells, including natural killer cells, macrophages/monocytes, peripheral B cells, and regulatory T cells, is decreased (Figure 2) 35, and the functions of antigen presentation and T-cell activation among aging dendritic cells, are also reduced (Figure 2).

The effect of senescence on carcinogenesis remains rather complex and controversial. As long as epithelial cells are in a state of senescence, their proliferation will be blocked, which prevents them from being regarded as tumor-derived cells. In contrast, they can also be regarded as storage pools for damaged cells, and once they re-enter the cell cycle, there is a risk of cancer-causing mutations (Figure 2). In addition, senescent hepatocytes secrete proinflammatory factors and matrix degradation molecules, which can stimulate the proliferation of cancer cells and epithelial-mesenchymal transformation. While these factors recruit immune-tolerant cells, they also attract immune cells to eliminate cancer cells 36.

The aged liver has a reduced ability to regenerate (Figure 2). Nikolai et al. reported that a polyprotein complex containing histone deacetylase 1 (HDAC1); SWI/SNF related, matrix-associated, actin-dependent regulator of chromatin, subfamily a, member 2 (BRM); and CCAAT/enhancer-binding protein (C/EBP)α was increased in aged hepatocytes (Figure 2), occupying E2F-dependent promoters and affecting the expression of genes about liver regeneration 37. In addition, a decline in growth hormone is associated with aging (Figure 2), so the reduced regenerative capacity of the liver is associated with the growth hormone axis 38. The two pathways are related because the decline in regeneration capacity can be attenuated by growth hormone in mice to remove the aforementioned HDAC1-C/EBPα-BRM inhibitory complex from the promoters 39. In fact, in our recent study, we also found that genes enriched in the cell cycle are downregulated and that E2F8, which is also downregulated in senescent hepatocytes, is the most significant transcription factor (TF) correlated with downregulated genes enriched in the cell cycle. In summary, when the capacity of hepatocyte regeneration declines, fewer cells enter S-phase in the aged liver.

### The current experimental model for research on liver aging

Presently, aged liver models are commonly used to study aging, and we review the published literature about liver aging models (Table 1). Hepatocyte aging models are induced by D-gal and H2O2, respectively, and for animal models, there are zebrafish models and D-gal-induced liver aging models. Additionally, aged liver samples from humans are also obtained to study the underlying mechanism of liver aging. In this section, we have divided these models into three distinct parts, namely, *in vitro* models, *in vivo* models, and human patient samples, and introduced them in detail.

### *In vitro* models

Seo et al. treated HepG2 cells and primary hepatocytes with H2O2 (500 µmol/L) for 48 hours and successfully induced cell aging 14. They then studied the changes in glucose and lipid metabolism and the accumulation of cholesterol in this model of aged hepatocytes 14. The results demonstrated that aged cells take up more cholesterol and glucose than younger cells and contributing to the synthesis of cholesterol from glucose, which leads to an accumulation of cholesterol in senescent hepatocytes 14. Additionally, Cong et al. used D-gal (200 mmol/L) to induce the senescence of hepatocytes for 120 hours 40. They reported that the levels of the aging markers cyclin-dependent kinase inhibitor 1A (P21) and P53 are increased and that SA-β-gal staining is positive in cells 40. Moreover, senescent hepatocytes have a decreased regeneration ability 40. Based on cell models, researchers can study the molecular mechanism of hepatocyte aging *in vitro*, and it is more convenient to screen antiaging drugs with this model than with others. Research findings can also be mutually validated in these two kinds of hepatocyte aging models induced by D-gal and H2O2, which increases the persuasiveness of the results.

### *In vivo* models

Many studies have reported the induction of liver aging by D-gal in rats 16, 41. Research on the mechanisms of D-gal-induced senescence, which include ROS production, oxidative stress induction, and increased inflammation, has been reported 16. These mechanisms all cause hepatocellular apoptosis, liver structural damage, and mitochondrial dysfunction 16. Dalia et al. evaluated the antiaging properties of sulforaphane on liver aging induced by D-gal (300 mg/kg*d, for 5 days a week) in rats 41. The results indicated that sulforaphane decreased serum levels of aspartate transaminase (AST), alanine transaminase (ALT), and total and direct bilirubin. It promoted hepatic antioxidant ability by regulating Kelch-like ECH-associated protein 1 (Keap-1), NF-E2-related factor 2 (Nrf-2), heme oxygenase-1 (HO-1), and antioxidant enzyme activity 41. In addition, sulforaphane can also decrease the levels of the inflammatory cytokines tumor necrosis factor-alpha (TNF-ɑ) and transforming growth factor-β (TGF-β) and inhibit hepatic fibrosis 41. Together, the data indicate that sulforaphane can inhibit D-gal-induced liver aging 41. Based on the D-gal-induced liver aging model, we can perform intervention studies on the liver *in vivo* to explore new treatments for liver aging and liver aging-related diseases.

In addition, Beatriz et al. demonstrated that recombination activating gene 1 mutation (rag1^-/-^) can accelerate the aging of zebrafish. Specifically, the senescence markers P21, P53, MDM2 proto-oncogene (MDM2), cyclin-dependent kinase inhibitor 1C (P57), and the activity of SA‐β‐gal are all increased in the livers of rag1^-/-^ zebrafish. Moreover, they also found that in the livers of rag1^-/-^ zebrafish, telomere length is reduced, and the ability to self-renew and repair is abnormal. These investigators suggested that L‐acetylcarnitine (ALCAR) could be considered a new biomarker for aging and is an essential metabolite for avoiding premature aging 42. In addition, ABT-263 (navitoclax) has a significant senolytic effect in Rag1^-/-^ zebrafish 42. Hence, Rag1^-/-^ zebrafish are a very useful model for studying the properties of liver aging *in vivo*, and researchers can use them to screen antiaging drugs. In addition, telomerase mutants can also accelerate the aging of zebrafish, and there are shorter telomeres in the telomerase-deficient zebrafish strain tert^hu3430/hu3430^ than in wild-type zebrafish 44. Similar to naturally aging zebrafish, tert^hu3430/hu3430^ zebrafish have degenerative phenotypes and liver dysfunction 44. Tert^hu3430/hu3430^ zebrafish more easily develop aging-related diseases than wild-type zebrafish and die prematurely 44. This telomerase mutant aging model can be used to unravel the relationship among telomere shortening, hepatic tissue regeneration, hepatic aging, and disease. Collectively, the evidence indicates that D-gal and zebrafish can be used to build an aging model and that animal models can be used to conduct further molecular studies on liver aging and screen effective antiaging drugs.

### Human patient samples

Currently, liver samples from humans are also used to study liver aging and liver aging-related diseases. The risk of hepatic fibrosis progression is highly increased when patients are infected with hepatitis viruses at an older age 45, 46. To understand the underlying mechanism, Norihisa et al. obtained liver samples from donors with normal livers and divided them into younger and older groups 46. They found that chitinase 3-like 1 (CHI3L1) was overexpressed in the aging liver 46. In addition, they obtained liver tissues from patients with liver cirrhosis and found that the expression level of CHI3L1 was increased in these cirrhotic livers 46. Next, they conducted a series of experiments and concluded that CHI3L1 played a central role in the increased susceptibility of aged livers to fibrosis progression 46. In addition, Raquel et al. first studied the hepatic function and microcirculatory status in aged livers from rats, and then they validated their research results in aged livers from humans 47. The results indicated that aged livers had dysfunctional hepatic sinusoids with increased vulnerability to injuries 47. The resistance of the hepatic vasculature and portal pressure were increased in the aged liver 47. Typical molecules from hepatic sinusoid cells were changed, and the function of hepatocytes also deteriorated in the aged liver 47. Moreover, hepatic macrophages were in a proinflammatory state, and hepatic stellate cells were activated spontaneously 47. Most importantly, these results were verified in aged liver samples from humans. Overall, these studies all used human liver specimens, increasing the reliability of the results. Researchers can find key molecules using aged human liver samples and then perform molecular experiments to study the characteristics of liver aging. Additionally, findings about liver aging from animal and cell models can be validated in aged liver specimens from humans directly or indirectly.

Indeed, patients older than 70 years old undergoing hepatectomy often show increased prevalence rates of comorbidities, and liver aging increases the incidence of graft failure after LT 48, 49. Thus, the above models provide opportunities for us to conduct deep data mining regarding liver aging to find the underlying targets. In doing so, aging models can be induced, and senescence markers (P16, P21, P53, SA‐β‐gal, and SASP) can be used to determine whether a model has been successfully constructed. Based on these models, we can perform basic and clinical studies to find key targets so that the quality of the aged liver can be improved.

### Omics analysis of the aged liver and potential mechanisms

Recently, omics analysis has gained momentum in liver aging. “Omics” refers to the acquisition of systematic knowledge of collective molecular features across the genome, transcriptome, proteome, metabolome, and microbiome. Omics data offer a potential way to depict the working environment of the liver in the body and are thus very helpful for mechanistic studies 50. In addition, comprehensive multi-omics studies have shown the advantages of improving the understanding of molecular functions and studying disease prediction, and they mutually validate each other at various levels 50. In Table 2, we provide a comprehensive review of omics studies related to liver aging. The search strategy for these studies is presented in Table S1 and the process of literature extraction is shown in Figure S1. Tabula Muris Senis data report differentially expressed genes in the aged livers of mice as determined by single-cell RNA sequencing 51. Ten senescence genes, bone marrow stromal cell antigen 1 (Bst1), cyclin-dependent kinase inhibitor 2A (Cdkn2a), E2F transcription factor 2 (E2f2), interleukin 10 (Il-10), interleukin 1 beta (Il-1b), integrin alpha M (Itgam), integrin alpha X (Itgax), lamin B1 (Lmnb1), poly (ADP-ribose) polymerase family, member 14 (Parp14), and tumor necrosis factor (Tnf), are overexpressed in the aged liver, especially in Kupffer cells 52. In addition, Han Qunhua et al. reported that the levels of 70 metabolites were decreased and that the levels of another 83 metabolites were increased in aged livers compared with young livers. Organic acids and derivatives, lipids and lipid-like molecules, organic oxygen compounds, and organoheterocyclic compounds were the top four ranked altered metabolites. Most of the metabolites with increased levels were glycerophospholipid metabolites and fatty acyl group members, especially lysoglycerophospholipids. Meanwhile, the levels of metabolites such as keto acids, carboxylic acids, and their derivatives were mostly decreased. Moreover, the differentially abundant metabolites were enriched mainly in the glycerophospholipid metabolic pathway 53. Regarding transcriptomic data, there are significant differences between young and old livers. Differential expression of genes in glycerophospholipid metabolism, arachidonic acid metabolism, histidine metabolism, and linoleic acid metabolism leads to changes in metabolites; for example, overproduction of phospholipase A2 (Pla2) catalyzes the formation of arachidonic acid and lysophospholipids from glycerophospholipids, which can cause hepatic disease and inflammation 54. In addition, histidine decarboxylase is overexpressed, which leads to increased production of histamine 55.

White et al. reported that aged mice show greater variability in the transcriptome of the liver than younger mice 56. Their study found that lymphocyte antigen 6 family member A (Ly6a), matrix metallopeptidase 12 (Mmp12), Cxcl9, guanylate binding protein 2 (Gbp2), interleukin 7 (Il7), Rac family small GTPase 2 (Rac2), fibroblast growth factor receptor 3 (Fgfr3), cathepsin S (Ctss), and telomerase RNA component (Terc) (noncoding RNA (ncRNA)) were overexpressed but that metallothionein 1 (Mt1), E2F transcription factor 7 (E2f7), heat shock protein 1B (Hspa1b), and nuclear paraspeckle assembly transcript 1 (Neat1) (ncRNA) were downregulated. In addition, these ncRNAs, Neat1, and Pvt1 oncogene (Pvt1) play important roles in the process of aging and maternally expressed gene 3 (Meg3), RNA imprinted and accumulated in nucleus (Rian), and miRNA containing gene (Mirg) all work together to regulate cell proliferation. They also reported that there were three main networks of liver aging: lipid metabolism, proliferative homeostasis, and inflammation. The network of inflammation is relevant to cancer because inflammatory cytokines secreted by aged cells can promote the hyperplastic growth of hepatocytes, which can cause an increase in aging-related tumors. Another network associated with aging involves the proliferation and death of hepatocytes. Proliferative homeostasis of the aged liver is lost, and the regeneration rate of hepatocytes is decreased 57. The third interaction network correlated with aging was the synthesis and oxidation of lipids. Cytochrome P450, family 8, subfamily b, polypeptide 1 (Cyp8b1); cytochrome P450, family 1, subfamily b, polypeptide 1 (Cyp1b1); cytochrome P450, family 4, subfamily a, polypeptide 14 (Cyp4a14); and cytochrome P450, family 26, subfamily a, polypeptide 1 (Cyp26a1) are the most abundant families of enzymes in the network. The failure of these pathways results in lipofuscin accumulation, which reflects a reduced ability of cells to metabolize their waste products 58.

Son et al. analyzed hepatic metabolites in aged rats. They found three kinds of metabolism associated with aging, including lipid energy metabolism through a betaine-methionine-carnitine system, degradation of nucleic acid metabolism, and nicotinamide adenine dinucleotide (NAD) metabolism. Although the levels of metabolites related to energy metabolism (betaine, methionine, carnitine, and fatty acylcarnitines) were increased, those of NAD and NAD/NADH were decreased. As a result, the production of energy did not increase by β-oxidation with aging because the activity of metabolism related to NAD declined with age. In addition, the production of nucleic acid degradation products (hypoxanthine and xanthine) increased with aging, which could have caused the accumulation of ROS. Therefore, these results strongly demonstrate that aging leads to the dysregulation of hepatic energy metabolism and the accumulation of ROS. The above results are helpful for us to better understand aging-related diseases 59.

**Figure 3:**
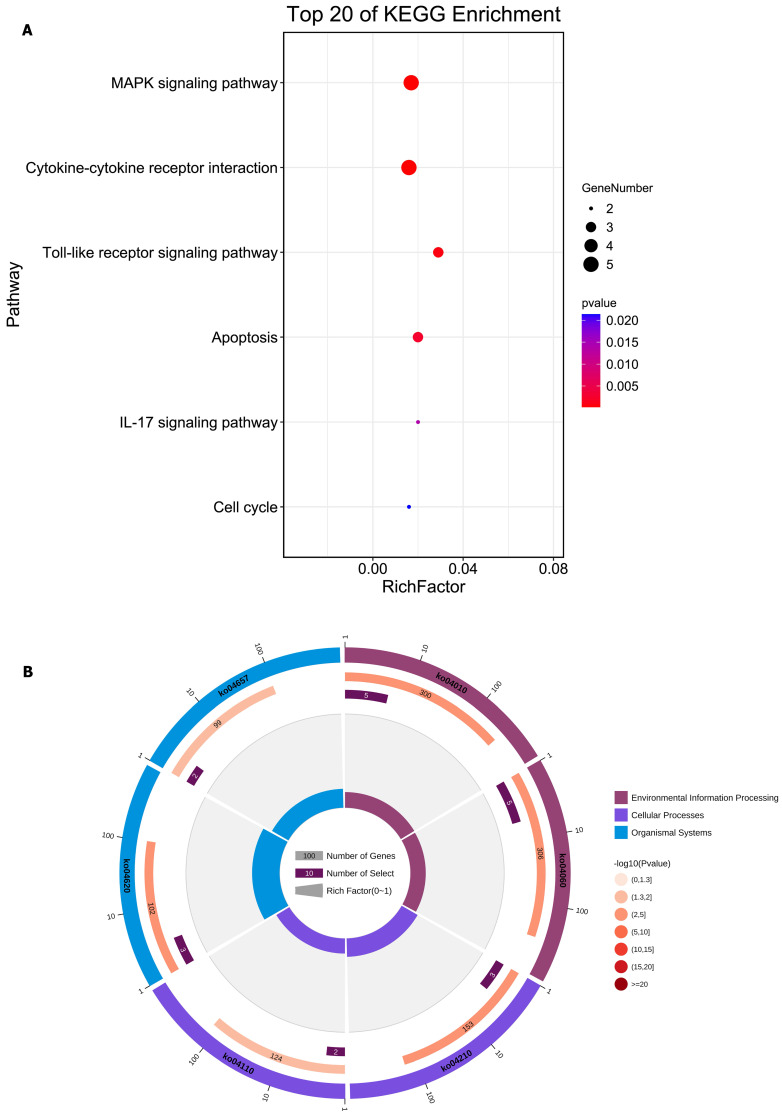
Reanalysis of positive genes associated with liver aging in prior omics studies.** (A)** Bubble diagram of enrichment analysis based on positive genes associated with the aged liver. **(B)** Circle diagram of enrichment analysis based on positive genes associated with the aged liver.

**Figure 4:**
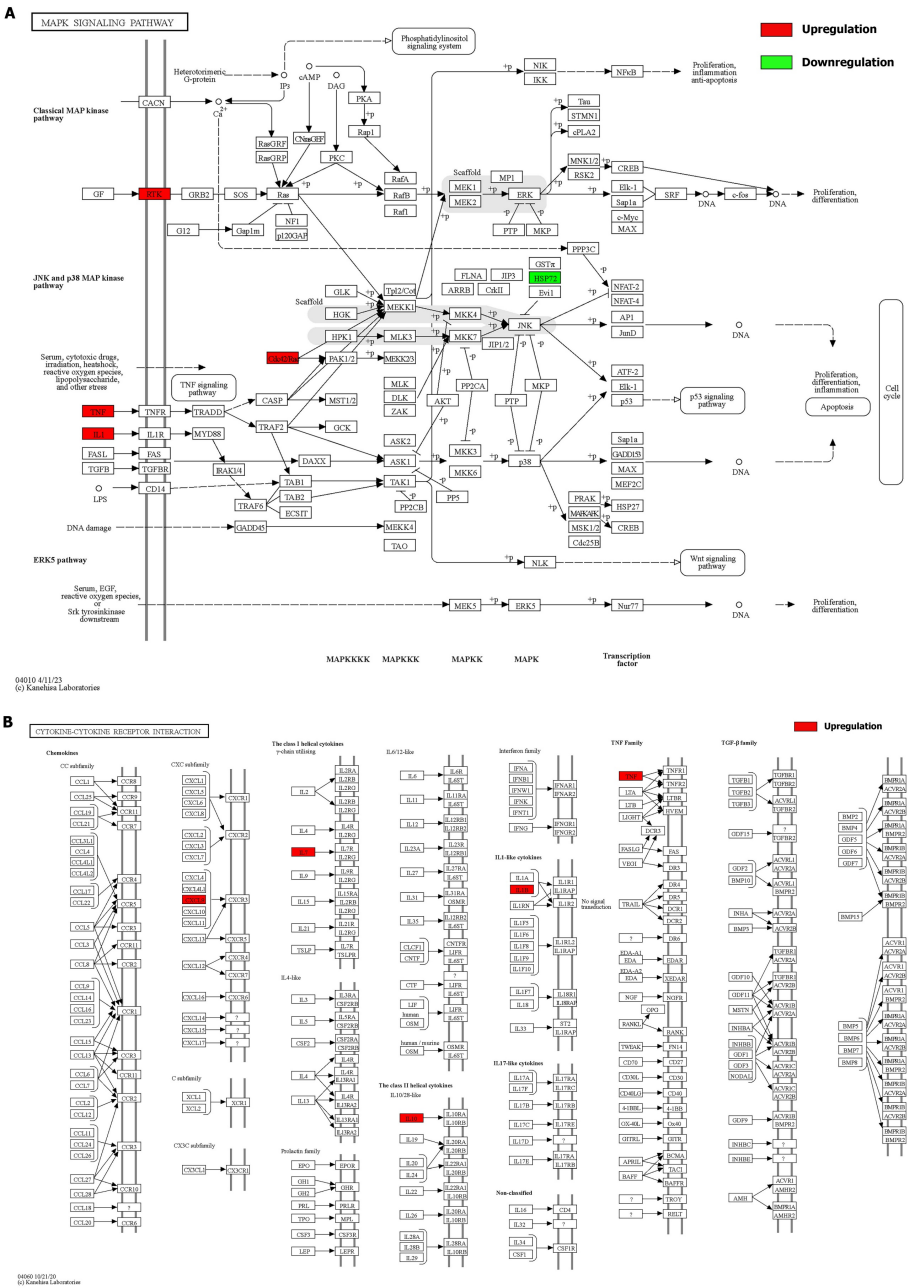
Details of positive signaling pathways and genes associated with liver aging. **(A)** Details of the MAPK signaling pathway and positive genes associated with liver aging. **(B)** Details of the cytokine-cytokine receptor interaction pathway and positive genes associated with liver aging.

**Figure 5:**
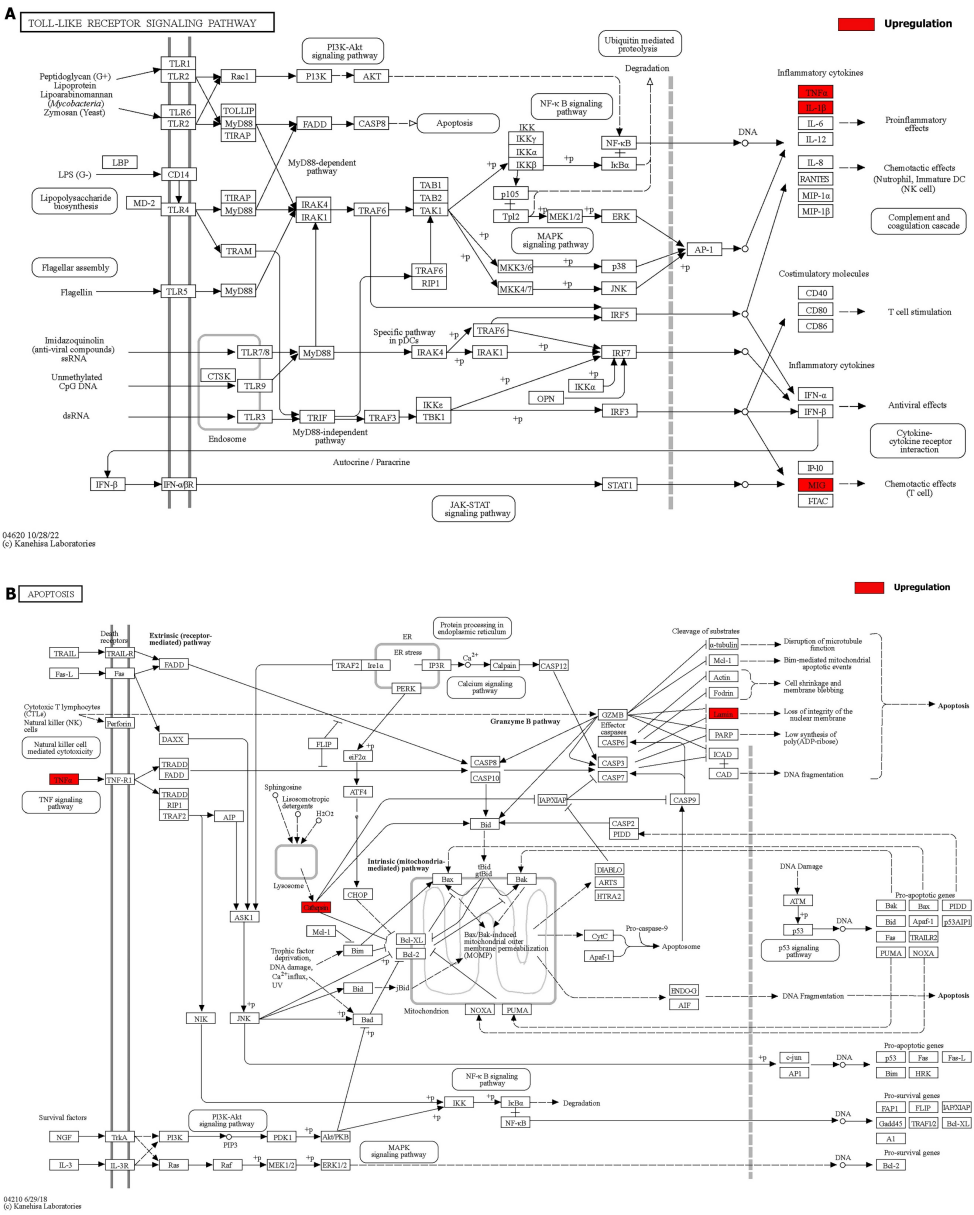
Details of positive signaling pathways and genes associated with liver aging. **(A)** Details of the toll-like receptor signaling pathway and positive genes associated with liver aging. **(B)** Details of the apoptosis pathway and positive genes associated with liver aging.

**Figure 6:**
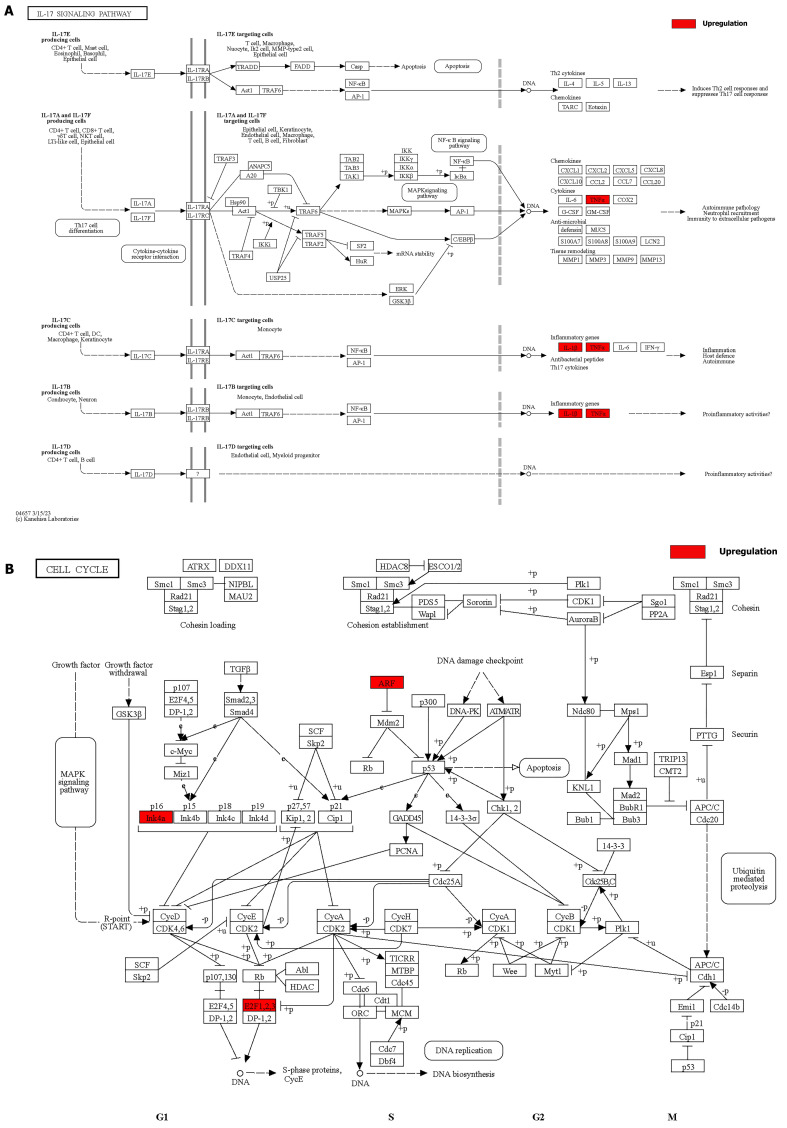
Details of positive signaling pathways and genes associated with liver aging.** (A)** Details of the IL-17 signaling pathway and positive genes associated with liver aging. **(B)** Details of the cell cycle pathway and positive genes associated with liver aging.

We integrated the different genes above and reanalyzed them using omics methods (Figure 3, Table 2). These results showed that the mitogen-activated protein kinase (MAPK) signaling pathway, cytokine-cytokine receptor interaction, toll-like receptor signaling pathway, apoptosis, interleukin 17 (IL-17) signaling pathway, and cell cycle pathway exhibited significant deviations in the aged liver (Figure 4-6). The MAPK signaling pathway plays an important role in liver aging and can regulate the expression of the senescence markers BCL2 apoptosis regulator (Bcl2), BCL2-associated X apoptosis regulator (Bax), and P21 60. In addition, the MAPK signaling pathway plays an important role in promoting the development of nonalcoholic fatty liver disease (NAFLD) 61, and aged livers are more susceptible than young livers to NAFLD. Cytokine‒cytokine receptor interactions, the toll-like receptor signaling pathway, and the IL-17 signaling pathway are all inflammation-related pathways and senescent hepatocytes secrete inflammatory factors contributing to liver aging-related diseases. For instance, Cxcl9 is overexpressed in the cytokine-cytokine receptor interaction pathway (Figure 4B). Cxcl9 promotes the senescence of cells, and silencing Cxcl9 can effectively reverse cellular senescence 62. The toll-like receptor signaling pathway also plays an important role in promoting liver fibrosis, and aged livers are generally susceptible to fibrosis 63. Hence, it is worth conducting further study on the toll-like receptor signaling pathway in the process of liver aging. In addition, senescent hepatocytes have compromised regenerative ability, and as shown in Figure 6B, the expression of cyclin-dependent kinase inhibitor P16 was increased. Overall, these pathways are all important in liver aging and worthy of further study.

There are some limitations in current liver aging-related omics studies. Overall, available omics data concerning liver aging are fragmented and lack systematic frameworks and validation tests. Moreover, most omics studies have been conducted using animal models; rarely have they used aged liver specimens from humans. Moreover, there is a lack of studies about whether there are any differences between commonly used animal models and humans. Next-generation sequencing and multi-omics studies with mutual validation in a fixed cohort are urgently needed in future liver aging-related omics investigations. In addition, more attention should be given to omics studies of blood samples, as blood can closely reflect liver aging. However, few studies including multidimensional samples (tissues and sera) have been conducted with liver aging models. These limitations may affect the translational value of omics data in liver aging research. However, there is an urgent need to use multi-omics approaches to study liver aging to discover key intervention targets. Thus, it is necessary to solve the above limitations in omics studies.

**Figure 7:**
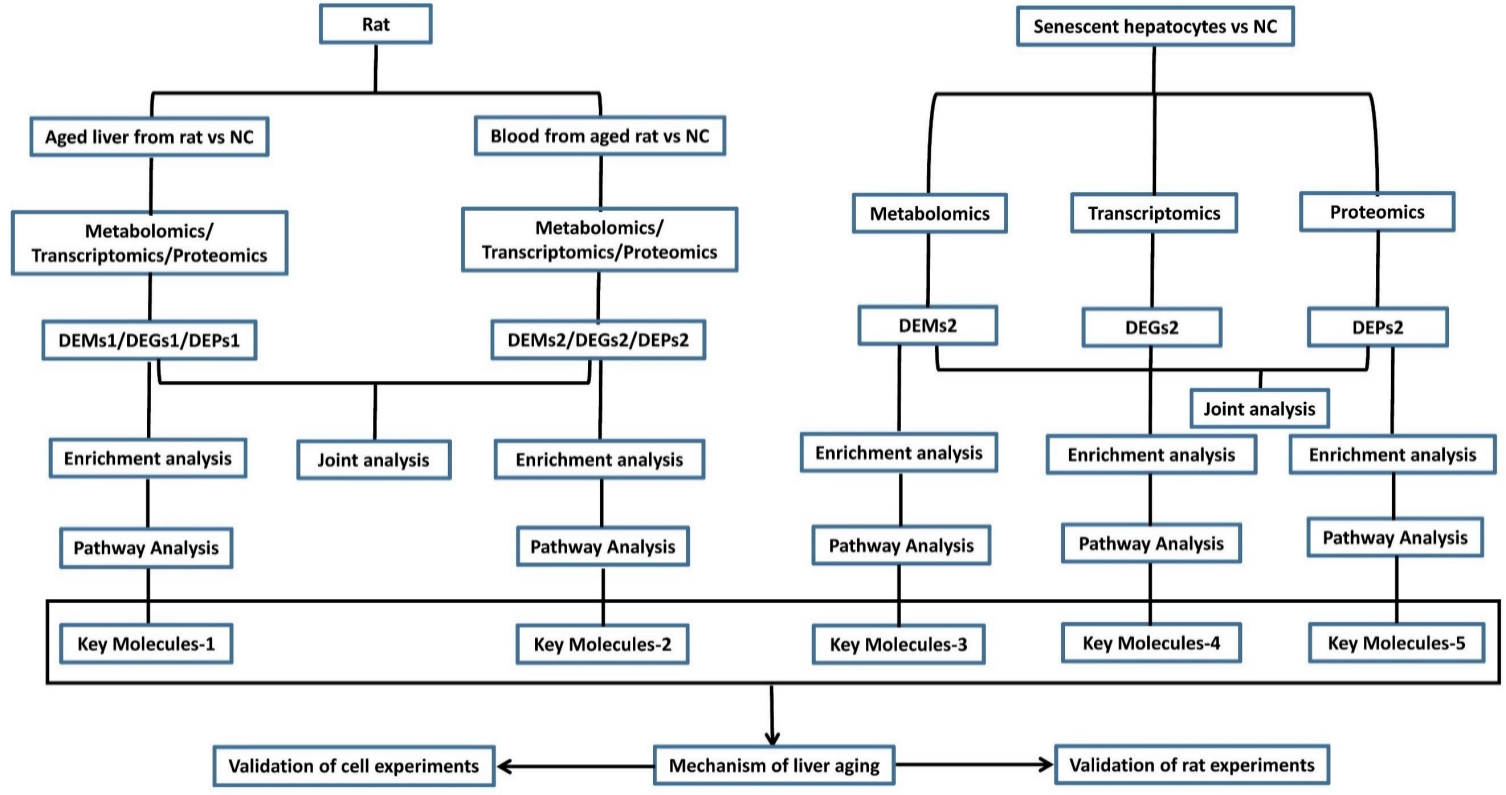
Flow diagram of the study design on liver aging. DEGs, differentially expressed genes; DEMs, differentially expressed metabolites; DEPs, differentially expressed proteins.

Hence, we formulated a flowchart of research on liver aging using multi-omics analysis and molecular experiments (Figure 7). The aged liver model can be natural or induced by D-gal, and senescence in hepatocytes can be induced by treatment of the HepG2 cell line with D-gal and H2O2, as reported in many studies 14, 64-66. Integrative transcriptomics, metabolomics, and proteomics analyses were performed on these blood, liver, and hepatocyte specimens. These joint multi-omics analyses depicted the panorama of the working environment for the liver in the body. The differentially expressed genes (DEGs), differentially expressed metabolites (DEMs), and differentially expressed proteins (DEPs) were used for enrichment pathway analysis, and we identified the most important molecules. According to reported studies on liver aging, we can speculate on the underlying mechanism and validate it with cell experiments and rat experiments.

### Potential therapeutic approaches and their application to liver aging

Antiaging is a promising and challenging field of research due to the complexity of aging mechanisms. Scientists have performed many anti-aging studies, including studies testing caloric restriction (CR) through diet control, clearance of senescent cells, and the treatment of liver aging with matrine 67, 68.

### Diet control

CR is effective against aging-related diseases and for improving physical condition. For instance, many research findings have demonstrated that CR can reduce the occurrence of obesity-induced aging-related diseases 69. In addition, moderate reductions in caloric intake help to improve the viability of organisms 70. Long-term CR without malnutrition can improve the efficiency of resting energy expenditure, which reduces oxidative damage to the liver and suppresses liver inflammation 71, 72. Miguel et al. reported that increasing the fasting time and reducing the total caloric intake are the determinants of prolonged longevity. Their multi-omics analysis indicated that the glycine-serine-threonine metabolic axis correlates with longevity and that short-chain fatty acid metabolism and polyunsaturated fatty acid metabolism is the key pathways for improving health. In addition, protein restriction in aging male mice is also beneficial for expanding lifespan and metabolic health, and fibroblast growth factor 21 (FGF21) is necessary for the antiaging effect of protein restriction 73. Dietary intervention and exercise can increase lipophagy to ameliorate high-fat diet-induced NAFLD and liver aging 74. The purposes of CR and exercise are to increase energy expenditure and reduce the burden on the body. In particular, a diet of low protein and low lipids may also be beneficial to liver metabolism and can expand longevity. In our opinion, lipid metabolism dysfunction is an important contributor to liver aging, and our recent study found that the pathways of fatty acid elongation, fatty acid degradation, and biosynthesis of unsaturated fatty acids are all downregulated in aged livers. Hence, the role of lipid metabolism in the liver needs to be further investigated. Overall, these findings provide us with new insight for understanding the remodeling of the genome/metabolome in the aged liver and will help design therapeutic interventions against aging-related metabolic alterations 75.

### Clearance of senescent cells

Presently, clearing senescent hepatocytes is a new strategy to protect against liver aging. For example, a newly developed drug type, known as a senolytic, can selectively clear senescent hepatocytes and maybe a new strategy to attenuate liver aging. Dasatinib (D) and quercetin (Q) can induce apoptosis in senescent hepatic progenitor cells and ameliorate aging-related phenotypes 76. Other senolytics, including A-1155463, A-1331852, ABT-737, and ABT-263, can also inhibit the BCL-2 family (BCL-W, BCL-XL, and BCL-2) and therapeutically target senescent hepatocytes 77. In addition, ATB263 can remove senescent cancer cells and hepatocytes with chemotherapy-induced senescence to inhibit the metastasis and recurrence of tumors 78, 79. Senolytics (D+Q) can also effectively remove P16-positive cells, reduce the activity of SA‐β‐gal, and limit the release of inflammatory factors 80, 81. Furthermore, Zhu et al. studied whether the effect of clearing senescent cells persists for many weeks after senolytics are no longer present, and the results demonstrated that D+Q treatment has a long-lasting effect after the drug is no longer present 76.

Although the proliferation of senescent cells is decreased, these cells are resistant to apoptosis. Thus, the prosurvival pathways can be regarded as targets to eliminate senescent cells by RNA interference. The survival viability of senescent hepatocytes can be decreased by interfering with the expression of ephrin B1 (EFNB1), ephrin B3 (EFNB3), phosphatidylinositol-4,5-bisphosphate 3-kinase delta catalytic subunit (PI3KCD), P21, BCL-xL or plasminogen-activated inhibitor-2 (PAI-2). In addition, forkhead box O4 (FOXO4) is pivotal in the viability of senescent hepatocytes because it can inhibit the apoptosis of senescent hepatocytes to maintain their activity. Hence, the FOXO4 peptide is a D-retro-inverso isoform induced by the modification of peptides, and it can disrupt the interaction between FOXO4 and P53 to induce the targeted apoptosis of senescent hepatocytes. Moreover, the FOXO4 peptide can cause nuclear exclusion of senescent cells. Hence, doxorubicin-induced senescence can be effectively reduced *in vitro* and *in vivo* by the FOXO4 peptide. In doing so, the doxorubicin-induced loss in body weight and liver toxicity can also be neutralized. The body can tolerate the FOXO4 peptide well, so it is a newly available option for clinicians to attenuate liver aging 27. Overall, clearing senescent hepatocytes can help to protect against liver aging and eliminate the adverse effects of senescent hepatocytes on peripheral normal hepatocytes.

### Matrine administration against liver aging

Matrine is an alkaloid extracted from Sophora flavescens that has a significant antiaging effect. It mainly inhibits cellular senescence and oxidative stress. First, matrine significantly inhibits the increase in aging-induced nuclear size in hepatocytes and inhibits the increase in SA-β-gal--positive hepatocytes. In fact, in our recent study, we also found that peroxisome protein expression was downregulated in aged livers and that catalase and malondialdehyde (MDA) levels also declined, which indicated that aged livers had decreased antioxidant ability. However, matrine can also inhibit the decline in the antioxidative ability of the aged liver; for instance, it can attenuate the decreases in total superoxide dismutase, catalase, and MDA. The expression of aging-related genes is also downregulated by matrine. P16, CDKN2D (P19), and P21 are all overexpressed in the aged liver, but matrine can significantly reverse their increases. Moreover, the expression levels of interleukin 1 beta (IL-1β) and interleukin 6 (IL-6) are reduced in senescent hepatocytes by matrine. Overall, the data indicate that matrine can be used to prevent liver aging and treat liver aging-related diseases 82.

## Conclusions

In summary, this review retrospectively reports the changes in the aged liver, cellular and animal models, and presents omics studies on liver aging. In addition, we discuss current treatments to prolong lifespan. Based on cell and animal models of liver aging, a multi-omics approach can be applied to find key metabolic pathways and target genes, and these pathways and targets can be validated through molecular experiments. Thus, in doing so, we can eliminate the adverse effects of liver aging to expand longevity and aged donor livers can be transplanted into recipients safely with the maintenance of homeostasis.

## Supplementary Material

Supplementary figure and table.

## Figures and Tables

**Table 1:** Basic information on the enrolled studies involving liver aging models.

Name, Publication year	Species, sample	Methods of inducing aging	Result	Clinical implications of aging model
Eunhui,2019	Human, HepG2 cells line, primary hepatocytes	H_2_O_2,_ 500 umol/L, 48h	ROS contributed to the accumulation of cholesterol in aged liver.	Based on cell models, researchers can study the molecular mechanism of hepatocyte aging *in vitro*, and it is more convenient to screen antiaging drugs with this model than with others. Research findings can also be mutually validated in these two kinds of hepatocyte aging models induced by D-gal and H2O2, which increases the persuasiveness of the results.
Cong,2014	Human, hepatocytes	D-gal,200mmol/L,120h	ROS and aging markers of P21, P53 and SA-β-gal were increased in hepatocytes. Moreover, senescent hepatocytes had decreased ability of regeneration.	
Dalia,2019	Rat, livers	D-gal, 300mg/kg*d, 30days	Sulforaphane regulated Keap-1, Nrf-2, HO-1 and antioxidant enzyme activities for reducing oxidative stress to against aging.	Based on the D-gal-induced liver aging model, we can perform intervention studies on the liver *in vivo* to explore new treatments for liver aging and liver aging-related diseases.
Khairunnuur,2021	Rat, livers	D-gal, 60-300mg/kg*d, 6-8weeks	D-gal induced the production of ROS and contributed to oxidative stress.	
Beatriz,2019	Zebrafish, livers	Rag1^-/-^	Rag1^-/-^ can accelerate the liver aging of zebrafish and ALCAR could be considered a new biomarker and target for aging.	Rag1-/- zebrafish are a very useful model for studying the properties of liver aging *in vivo*, and researchers can use them to screen antiaging drugs.
Madalena,2016	Zebrafish, livers	Telomerase mutant	Telomerase mutant can construct the aging model for the study of physiological aging. Similar to natural zebrafish aging, tert^hu3430/hu3430^ zebrafish had degenerative phenotypes and dysfunctional liver. It developed aging related diseases more easily and died prematurely	Telomerase mutant aging model can be used to unravel the relationship among telomere shortening, hepatic tissue regeneration, hepatic aging, and disease.
Norihisa, 2021	Human, livers	Nature aging	CHI3L1 played a central role in the increased susceptibility of aged livers to fibrosis progression.	Researchers can find key molecules using aged human liver samples and then perform molecular experiments to study the characteristics of liver aging. Additionally, findings about liver aging from animal and cell models can be validated in aged liver specimens from humans directly or indirectly.
Leonardo, 2021	Human, livers	Nature aging	This study indicated that in aged liver, hepatic sinusoid was significantly dysfunctional and vulnerable to injuries.	

H_2_O_2_, hydrogen peroxide; ROS, reactive oxidative species; D-gal, D-galactose; P21, cyclin dependent kinase inhibitor 1A; P53, tumor protein p53; SA-β-gal, senescence-associated beta-galactosidase; Keap-1, Kelch-like ECH-associated protein-1; Nrf-2, NF-E2-related factor-2; HO-1, hemeoxygenase-1; Rag1**^-/-^**, recombination activating gene 1 mutants; ALCAR, L‐acetylcarnitine.

**Table 2:** Basic information on the enrolled omics studies on liver aging.

Name, Publication year	Species, sample, assay	Key molecule	Key pathway
Tabula, 2019	Mice, liver, single-cell transcriptomics	UP: Bst1, Cdkn2a, E2f2, Il-10, Il-1b, Itgam, Itgax, Lmnb1, Parp14, Tnf	Na
Qunhua, 2021	Rat, liver, metabolomics, transcriptomics	Metabolites-UP: glycero-phospholipid metabolites, fatty acyl, lysoglycerophospholipids; Metabolites-DN: keto acids, carboxylic acids; transcriptomics-up: pla2, arachidonic acid, lysophospholipids; histidine decarboxylase, histamine	Glycerophospholipid metabolic pathway, glycerophospholipid metabolism, arachidonic acid metabolism, histidine metabolism and linoleic acid metabolism
Ryan, 2015	Mouse, liver, transcriptomics	UP: Ly6a, Mmp12, Cxcl9, Gbp2, Il7, Rac2, Fgfr3, Ctss, and Terc(ncRNA); DN: Mt1, E2f7, Hspa1b, and Neat1(ncRNA);	Lipid metabolism, proliferative homeostasis, inflammation
Nari, 2012	Rat, liver, metabolomics	UP: betaine, methionine, carnitine, fatty acylcarnitines, hypoxanthine and xanthine, ROS; DN: NAD, NAD/NADH	lipid energy metabolism, degradation of nucleic acid metabolism, NAD metabolism

## Abbreviations

Cxcl9: C-X-C motif chemokine ligand 9
P16: cyclin dependent kinase inhibitor 2A
P53: tumor protein p53
E2F8: E2F transcription factor 8
TFDP1: transcription factor Dp-1
ROS: reactive oxygen species
H_2_O_2_: hydrogen peroxide
D-gal: D-galactose
LT: liver transplantation
IRI: ischemia-reperfusion injury
SA-β-gal: senescence-associated beta-galactosidase
SAHF: senescence associated heterochromatic foci
SADF: senescence-associated DNA damage foci
SASP: senescence-associated secretory phenotype
HDAC1: histone deacetylase 1
BRM: SWI/SNF related, matrix associated, actin dependent regulator of chromatin, subfamily a, member 2
C/EBP: CCAAT/enhancer-binding protein
TF: transcription factor
Rag1^-/-^: recombination activating gene 1 mutants
P21: cyclin dependent kinase inhibitor 1A
MDM2: MDM2 proto-oncogene
P57: cyclin dependent kinase inhibitor 1C
ALCAR: L‐acetylcarnitine
Bst1: bone marrow stromal cell antigen 1
Cdkn2a: cyclin dependent kinase inhibitor 2A
E2f2: E2F transcription factor 2
Il-10: interleukin 10
Il-1b: interleukin 1 beta
Itgam: integrin alpha M
Itgax: integrin alpha X
Lmnb1: lamin B1
Parp14: poly (ADP-ribose) polymerase family, member 14
Tnf: tumor necrosis factor
Pla2: phospholipase A2
Ly6a: lymphocyte antigen 6 family member A
Mmp12: matrix metallopeptidase 12
Gbp2: guanylate binding protein 2
Il7: interleukin 7
Rac2: Rac family small GTPase 2
Fgfr3: fibroblast growth factor receptor 3
Ctss: cathepsin S
Terc: telomerase RNA component
DN: downregulation
Mt1: metallothionein 1
E2f7: E2F transcription factor 7
Hspa1b: heat shock protein 1B
Neat1: nuclear paraspeckle assembly transcript 1
Pvt1: Pvt1 oncogene
Meg3: maternally expressed gene 3
Rian: RNA imprinted and accumulated in nucleus
Mirg: miRNA containing gene
Cyp8b1: cytochrome P450, family 8, subfamily b, polypeptide 1
Cyp1b1: cytochrome P450, family 1, subfamily b, polypeptide 1
Cyp4a14: cytochrome P450, family 4, subfamily a, polypeptide 14
Cyp26a1: cytochrome P450, family 26, subfamily a, polypeptide 1
NAD: nicotinamide adenine dinucleotide
MAPK: mitogen-activated protein kinase
IL-17: interleukin 17
Bcl2: BCL2 apoptosis regulator
Bax: BCL2 associated X, apoptosis regulator
NAFLD: nonalcoholic fatty liver disease
DEGs: differentially expressed genes
DEMs: differentially expressed metabolites
DEPs: differentially expressed proteins
CR: calorie restriction
FGF21: fibroblast growth factor 21
D: Dasatinib
Q: Quercetin
EFNB1: ephrin B1
EFNB3: ephrin B3
PI3KCD: phosphatidylinositol-4,5-bisphosphate 3-kinase delta catalytic subunit
PAI-2: plasminogen-activated inhibitor-2
FOXO4: forkhead box O4
MDA: malondialdehyde
P19: CDKN2D
IL-1β: interleukin 1 beta
IL-6: interleukin 6
