# Supplementary material for: The biological mechanism and emerging therapeutic interventions of liver aging
Source: Int J Biol Sci. 2024 Jan 1;20(1):280–95. doi: 10.7150/ijbs.87679 (PMC10750291; doi:10.7150/ijbs.87679)

Table S1 Literature search strategy across different databases.

ISI-web of science	Indexes=SCI-EXPANDED, SSCI, A&HCI, CPCI-S, CPCI-SSH, ESCI, CCR-EXPANDED, IC Timespan=1980-2018	
#1	(TS= (Liver aging)) AND DOCUMENT TYPES:(Article)	396326
#2	(TS= (Aged liver)) AND DOCUMENT TYPES:(Article)	396326
#3	(TS= (Old liver)) AND DOCUMENT TYPES:(Article)	115232
#4	(TS= (Older liver)) AND DOCUMENT TYPES:(Article)	115076
#5	#1 OR #2 OR #3 OR #4	452131
#6	(TS= (Transcriptomics)) AND DOCUMENT TYPES:(Article)	39680
#7	(TS=(Metabolomics)) AND DOCUMENT TYPES: (Article)	67934
#8	#6 OR #7	101607
#9	(TS= (Rat)) AND DOCUMENT TYPES:(Article)	2701243
#10	(TS= (Mouse)) AND DOCUMENT TYPES:(Article)	2757352
#11	#9 OR #10	4954678
#12	#5 AND #8 AND #11	488
Embase		
#1	'Liver aging' AND [article]/lim	19917
#2	'Aged liver' AND [article]/lim	246117
#3	'Old liver' AND [article]/lim	84993
#4	'Older liver' AND [article]/lim	14690
#5	#1 OR #2 OR #3 OR #4	318627
#6	'Transcriptomics' AND [article]/lim	52117
#7	'Metabolomics' AND [article]/lim	53307
#8	#6 OR #7	101341
#9	'Rat' AND [article]/lim	1732958
#10	'Mouse' AND [article]/lim	1774342
#11	#9 OR #10	3353746
#12	#5 AND #8 AND #11	470
PubMed		
#1	Search liver aging Filters: Full text	16924
#2	Search aged liver Filters: Full text	202456
#3	Search old liver Filters: Full text	58959
#4	Search older liver Filters: Full text	11138
#5	(((liver aging OR aged liver) OR old liver) OR older liver) Filters: Full text	25496
#6	Search transcriptomics Filters: Full text	298361
#7	Search metabolomics Filters: Full text	75062
#8	Search (transcriptomics OR metabolomics) Filters: Full text	362517
#9	Search rat Filters: Full text	1416854
#10	Search mouse Filters: Full text	1744574
#11	Search (rat OR mouse) Filters: Full text	2989866
#12	(((liver aging OR aged liver) OR old liver) OR older liver) AND (transcriptomics OR metabolomics) AND (rat OR mouse) Filters: Full text	1212

Figure S1 The process of literature extraction for enrolled studies.

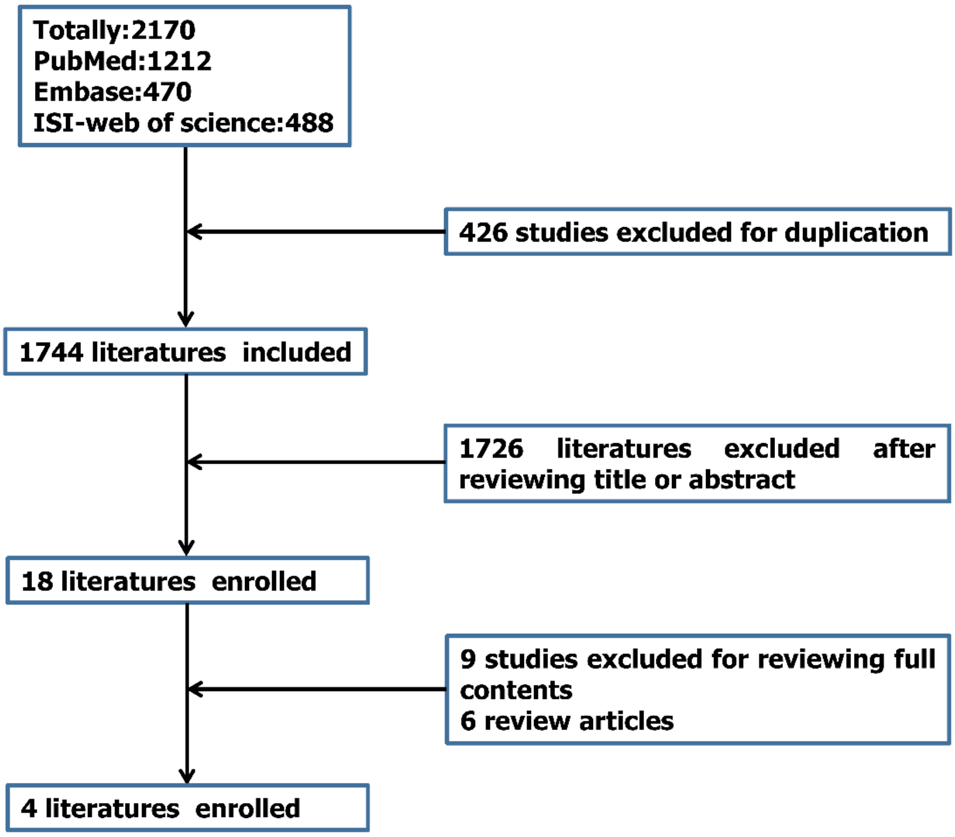

Supplement: Supplementary file 1 — Supplementary figure and table. [file ijbsv20p0280s1.pdf]
